# The Vitamin D, IL-6 and the eGFR Markers a Possible Way to Elucidate the Lung–Heart–Kidney Cross-Talk in COVID-19 Disease: A Foregone Conclusion

**DOI:** 10.3390/microorganisms9091903

**Published:** 2021-09-07

**Authors:** Mario Giosuè Balzanelli, Pietro Distratis, Rita Lazzaro, Angelo Cefalo, Orazio Catucci, Sergey Khachatur Aityan, Gianna Dipalma, Luigi Vimercati, Alessio Danilo Inchingolo, Maria Elena Maggiore, Antonio Mancini, Luigi Santacroce, Loreto Gesualdo, Van Hung Pham, Donatello Iacobone, Maria Contaldo, Rosario Serpico, Antonio Scarano, Felice Lorusso, Tran Cong Toai, Silvio Tafuri, Giovanni Migliore, Angelo Michele Inchingolo, Kieu Cao Diem Nguyen, Francesco Inchingolo, Diego Tomassone, Ciro Gargiulo Isacco

**Affiliations:** 1 SET-118, Department of Pre-Hospital and Emergency, SG Giuseppe Moscati Hospital, 74121 Taranto, Italy; mario.balzanelli@gmail.com (M.G.B.); distratispietro@gmail.com (P.D.); rita-lazzaro@libero.it (R.L.); ugemel@yahoo.it (A.C.); oraziocatucci@live.it (O.C.); 2 Director Multidisciplinary Research Center, Lincoln University, Oakland, CA 94612, USA; aityan@lincolnuca.edu; 3 Department of Interdisciplinary Medicine (D.I.M.), University of Bari “Aldo Moro”, 70124 Bari, Italy; giannadipalma@tiscali.it (G.D.); luigi.vimercati@uniba.it (L.V.); ad.inchingolo@libero.it (A.D.I.); m.e_maggiore@yahoo.it (M.E.M.); dr.antonio.mancini@gmail.com (A.M.); angeloinchingolo@gmail.com (A.M.I.); drkieukaren@gmail.com (K.C.D.N.); francesco.inchingolo@uniba.it (F.I.); 4 President, School of Medicine, University of Bari, 70100 Bari, Italy; loreto.gesualdo@uniba.it; 5 Department of Microbiology, Phan Chau Trinh University of Medicine and Nam-Khoa Biotek, Ho Chi Minh City 70000, Vietnam; van.pham@pctu.edu.vn; 6 SET-118, Department of Pre-Hospital and Emergency, BAT, 70100 Bari, Italy; donato.iacobone@aslbat.it; 7 Multidisciplinary Department of Medical-Surgical and Dental Specialties, University of Campania Luigi Vanvitelli, Via Luigi de Crecchio, 6, 80138 Naples, Italy; maria.contaldo@unicampania.it (M.C.); rosario.serpico@unicampania.it (R.S.); 8 Department of Innovative Technologies in Medicine and Dentistry, University of Chieti-Pescara, 66100 Chieti, Italy; ascarano@unich.it; 9 Department of Histology-Embryology and Genetic, “Pham Ngoc Thach”—University of Medicine, Ho Chi Minh City 70000, Vietnam; trancongtoai@pnt.edu.vn; 10 Department of Biomedical Science and Human Oncology, University of Bari, 70100 Bari, Italy; silvio.tafuri@uniba.it; 11 University Hospital of Bari, 70124 Bari, Italy; giovanni.migliore@policlinico.ba.it; 12 Department of Regenerative medicine, American Stem Cells Hospital, Ho Chi Minh City 70000, Vietnam; 13 HOLOS Medical and Research Center, 00071 Pomezia, Italy; dietomoh@gmail.com; 14 Foundation of Physics Research Center, 87053 Celico, Italy

**Keywords:** vitamin D, eGFR, IL-6, SARS-CoV-2, Coronavirus 19 (COVID-19), lung–heart–kidney cross-talk

## Abstract

Background: Based on recent findings, we speculated the existence of the lung, heart, and kidney axis as the main pathway for the COVID-19 disease progression. Methods: This paper reports on an observational study conducted by a team of researchers and doctors of the 118-Pre-Hospital and Emergency Department of SG Moscati of Taranto City in Italy. The study was conducted on a totality of 185 participants that were divided into three groups. The study group included COVID-19 affected patients (PP *n* = 80), the first control group included patients with different pathologies (non-COVID-19 NNp *n* = 62) of the SG Moscati Hospital, and the second control group included healthy individuals (NNh *n* = 43). The core of the current trial was focused on assessing the level of the vitamin D (serum 25(OH) D concentration), IL-6, and the renal glomerular filtrate (eGFR) in COVID-19 disease and non-COVID-19 patients in both groups. Results: It was observed that the majority of COVID-19-infected patients showed a progressive multi-organ involvement, especially in regard to the lung, kidney, and heart. The majority of the COVID-19 patients exhibited preexisting comorbidities which include cardiovascular, respiratory, and renal disorders accompanied by a severely low level of vitamin D, extremely high level of IL-6, and low glomerular filtration rate (eGFR). The significant overall damages exerted by the immune-mediated responses under the hyper-expression of proinflammatory cytokines and interleukins, such as IL-6, may be facilitated by either a decreased level of vitamin D or the ageing process. The reduced presence of vitamin D was often found together with a reduced functionality of renal activity, as revealed by the low eGFR, and both were seen to be concomitant with an increased mortality risk in patients with lung disorders and heart failure (HF), whether it is showed at baseline or it develops during manifestation of COVID-19. Therefore, the documentation of the modifiable risk factors related to SARS-CoV-2 and lung impairment in older patients with kidney and heart disease may help the clinician to better manage the situation. Conclusions: This paper addresses how a low level of vitamin D and older age may be indicative of systemic worsening in patients with COVID-19, with a goal of providing a broader context in which to view a better therapeutic approach.

## 1. Introduction

This paper recounts an observational study conducted by a team of researchers and doctors of the 118-Pre-Hospital and Emergency Department of SG Moscati of Taranto City in Italy. The study included a totality of 185 participants composed by males and females from 20 to 103 years old. The participants were divided into three groups. The study group included COVID-19 affected patients (PP *n* = 80; males *n* = 46; females *n* = 36; median age = 53.5); the first control group included patients with different pathologies (NNp *n* = 62; males *n* = 32; females *n* = 30; median age = 62) of the SG Moscati Hospital, and the second control group included healthy individuals (NNh *n* = 43; males *n* = 33; females *n* = 10; median age = 55). The inclusion criteria for the PP group admitted to 118 Unit were based on clinical and objective symptoms (coughing, fever, dyspnea, positive to nasal–pharyngeal swab (RT-PCR), and thoracic CT scan with ground glasses opacities). Meanwhile, the NNp and NNh groups were composed of individuals randomly chosen. The NNp individuals were out-patients suffering from different types of respiratory illness, i.e., mainly chronic obstructive pulmonary disease (COPD). The NNh group included voluntary healthy individuals from the same area.

The core of the current trial was focused on assessing the level of the vitamin D (serum 25(OH)D concentration), IL-6, and the renal glomerular filtrate (eGFR) in COVID-19 disease and non-COVID-19 patients in both groups. It was observed that the majority of COVID-19-infected patients showed a progressive multi-organ involvement, especially the lung, kidney and heart. The pathophysiology that underlies the complex loop of lung–heart–kidney interactions is a well-accepted assumption, a kind of respiratory–cardio–renal syndrome condition that gives rise to a susceptibility to many primary and secondary dysfunctions, such as infections, auto-immune reactions, sepsis, and declined endocrine activity (low level of erythropoietin (EPO)). In COVID-19 patients, there is often seen a long-term clinical history of kidney/heart deficit advocated by a compromised renin angiotensin system (RAS) [1,2,3,4,5,6,7,8,9,10,11].

In this case, the lung–kidney–heart cross-talk assumption could explain simultaneously the whole complexity of COVID-19 disease and its progression mechanism and the hysterical autoimmune activities under the guidance of the IL-6 which that leads to the notorious “cytokine storm” [1,2,3,4,5,12,13,14,15]. Findings from several observational studies reported that decreased level of vitamin D, in a concentration lower than 20 ng/mL appraised in COVID-19 patients, could be correlated to the worsening of the disease; on the other hand, a low concentration of vitamin D was considered a high risk of community-acquired pneumonia in relation to its role in suppressing autoimmune hyper-responses [16,17,18].

Still, a matter of great debate whether this dysfunction would be solely a matter of vitamin D—otherwise known as pro-hormone D—deficiency or a combined condition which ultimately involves kidney, heart and lung functions as well. As lungs capacity lowers due to a drop of microvascular homeostasis, heart functionality also worsens, and a compromised reuptake of filtered 25-hydroxyvitamin D in kidney proximal tubules may be one of the major events within this negative loop. Nevertheless, the vitamin D that has endocrine, paracrine, and autocrine functions also works as an functional inhibitor of the RAS, due to its involvement in preventing the angiotensin II (Ang-II) accumulation through the inhibitory activity on renin release, an event commonly experienced in patients infected with COVID-19 [1,2]. It is well certified the role of Ang-II in cholesterol plaque accumulation along arteries, veins, and visceral glomerular epithelial cells (podocytes) by regulating the expression of cholesterol metabolism-related molecules and that the subsequent cholesterol metabolism dysfunction resulted in kidney and cardio-vascular injuries [1,2,3]. In addition, corroborated evidence from experimental studies conducted on both animal models and in patients has indicated Ang II involvement in the pathophysiology of cardiac hypertrophy and failure, a conclusion based on the following observations: (1) Ang-II is produced within the myocardium, (2) Ang-II is activated within the failing hypertrophic heart, and (3) the pharmacological inhibition of the RAS and Ang-II in animal models and in patients with a hypertrophic heart experiencing a myocardial failure showed to be highly efficacious [4].

Nonetheless, does vitamin D therapy eventually solve the problem, and would the D treatment reverse the entire condition?

A priori, we should highlight the “pivotal role” of vitamin D within the immune system, either in preventing serious infections or in contributing to reducing the grade of severity of a disease. First, vitamin D deficiency is a very common phenomenon worldwide according to many studies and researches. For instance, vitamin D deficiency <30 mg/mL is present in 30–60% of the populations of Western, Southern, and Eastern Europe and in up to 80% of populations in Middle-Eastern countries. Secondly, even more severe deficiency (serum levels <15 mg/mL) has been reported in over 10% of Europeans [1]. Finally, D deficiencies that are considered in a range inferior to 45/50 ng/mL tend to go completely unnoticed and completely obfuscate the real need, which is between 50 and 80 ng/mL, for those individuals living in big cities and centers [1,2].

Furthermore, as the vitamin D receptor (VDR) is expressed on immune cells, such as the T–B lymphocytes and antigen presenting cells (APCs) (macrophages and dendritic cells), it follows that these cells are also capable of synthesizing the active vitamin D metabolite, a trait that confirms the vitamin D biological capacity to perform in an autocrine/immune fashion. It follows that the vitamin D ability of exerting a powerful immune modulatory activity takes place in several ways: (1) By the inhibition of the transcription of T-helper (Th) 1 proinflammatory cytokines and by enhancing the modulating activity of Th2 and M2 by lowering the secretion of interferon gamma (IFN)-γ, IL-17, IL-2, and IL-21 by enhancing CD4^+^ T cells activity; and (2) by inducing T-regulatory lymphocytes (Tregs− CD4 + CD25+). Of note, the Treg levels have been reported to be low in COVID-19 patients, and markedly lower in severe cases of respiratory viral disease, suggesting that, if Treg levels could be increased, this might be beneficial for diminishing the severity of viral attack and COVID-19 intensity. On the other hand, it is well accepted that a low level of vitamin D is an extensively experienced situation and is often reminiscent of significant exposure to upper respiratory tract infections [19,20,21,22,23,24,25].

Of note, in COVID-19 units and hospitals, it is a very common occurrence to see severe vitamin D deficiency and low eGFR associated with an increased level of IL6 as risk of SARS-CoV-2 infection progression and a poor prognosis. Thus, we speculated that, in COVID-19, uncontrolled IL-6 elevation and decreased eGFR may just reflect a state of immunity complication that could be somehow explained by vitamin D depletion [26,27,28,29] Our conclusion confirmed by the analysis performed on Covid-19-infected adults revealed an anomalous immunity picture composed of abnormal levels of both innate and adaptive immune components, such as monocyte, macrophages, and neutrophil, as well as CD4 and CD4-naïve T cells and B cells (data not shown). Patterns eventually reflected further complications that involved the CO_2_/O_2_ gas-exchange mechanism and the pH balance. The performed arterial blood gas (ABG) showed, for the majority of COVID-19 patients, an alkalotic pH accompanied by hypoxia (low O_2_) and hypocapnia (low CO_2_) condition, suggestive of a compromised cardio, lung, and kidney axis. The overall picture may eventually help to explain the particular involution of some of COVID-19 patients who ended up with sudden severe respiratory and cardiac complications and unexpected deaths [30,31,32,33]. 

Accordingly, in this current study, we assessed and compared the vitamin D, IL-6, and eGFR levels in serum obtained from three different groups, namely a COVID-19 patients group (PP *n* = 80) and a healthy (NNp *n* = 62) group and non-healthy (NNh *n* = 43) group used as controls (total *n* = 185). Accordingly, in this current study, the aim was to assess the vitamin D, IL-6, and eGFR levels in serum obtained from three different groups of COVID-19 patients.

## 2. Materials and Methods

### 2.1. Study Design

The present study on SARS-CoV-2/COVID-19 was conducted by the 118 Pre-Hospital and Emergency Department of the SG Moscati of Taranto Province Italy. The protocol was approved by the Institutional review boards of the SG Moscati Hospital and ASL Taranto, and all the participants were provided of an informed consent. 

Clinical data, including all medications and diagnoses, were extracted from medical charts and entered into a single database. Additional data were collected through study-specific physical examinations, laboratory testing, and an assisted interview.

### 2.2. Specimen Collection and Laboratory Measurements

At the entry, whole-blood and plasma specimens in EDTA were collected and given to the SG Moscati laboratory for the specific required analysis. All biomarkers were measured on the study entry specimens. Clinical site laboratory testing included measurement of total CBC, eGFR, urea, uric acid, creatinine, glucose, bilirubin (total, direct and indirect), sodium, potassium, chloride, calcium, AST, ALT, GGT, LDH, CK, lipase, amylase, troponin, CRP, VES, D-dimer, fibrinogen, procalcitonin, INR, aPTT, 25OH vitamin D, and IL6. Vitamin D was measured as 25(OH)D with the 25(OH)D iodine 125 radioimmunoassay (Diasorin, Saluggia, Italy); the IL-6 was measured by using an enzyme-linked immunosorbent assay (ELISA) based assay.

### 2.3. Statistical Methods

In the present study, three groups were evaluated: COVID-19 patients group (PP *n* = 80), and a healthy (NNp *n* = 62) group and non-healthy (NNh *n* = 43) group used as controls (total *n* = 185). The current study was performed during a period of seven months, between April and October 2020, and we used weighted linear regression to assess the relationship between prevalence of vitamin D deficiency, low eGFR, and high IL-6 and COVID-19 incidence in all individuals. To the best of our knowledge, this is one of the few studies that have investigated the role of vitamin D in the pathogenesis and treatment of COVID-19 in a clinical setting. In our population, we could observe major differences in COVID-19 and non-COVID in regard to the level of vitamin D, eGFR, and IL-6. The hypotheses about differences of the mean values of vitamin D, IL-6, and eGFR levels in PP, NNp, and the control NNh groups were tested by using the Student’s *t*-test with the significance level alpha = 5% (confidence level 95%).

At the study entry, D levels were categorized as follows: normal (≥50 ng/mL), sufficient (35–49 ng/mL), deficient (<35 ng/mL), or severe deficient (<20 ng/mL), as per the Italian Ministry of Health and United States Endocrine Society guidelines. The the IL6 was categorized as normal (<7 pg/mL), medium severe (8–20 ng/mL), and severe (≥20 pg/mL). The eGFR was categorized as normal (90–135 mL/min) [2]. Study entry characteristics were summarized with frequencies or medians and interquartile ranges (IQRs), as appropriate. All data were compared among the three groups, matching the vitamin D, eGFR, and IL6 levels by using the Student’s *t*-tests (assessing both Z and T score). The relative percent differences between those with deficient versus non-deficient vitamin D, IL6, and eGFR levels were assessed with general linear models. Odds ratios expressing the risk of vitamin D deficiency per doubling of the biomarker were calculated with their corresponding 95% confidence intervals. Multivariable models were adjusted for baseline variants that differed between vitamin D, eGFR, and IL6 groups: age and gender since COVID-19 diagnosis.

## 3. Results

### 3.1. Clinical Characteristics of the Research Subjects

**Figure 1:**
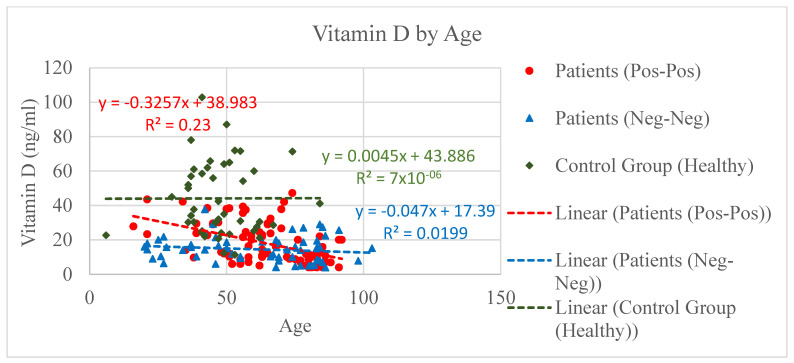
Scatter charts and regression lines for vitamin D levels by age for COVID-positive (PP) and COVID-free patients (NNp and healthy group NNh). The level of vitamin D is higher in the NNh group if compared to both PP and NNp. Of note, though both the PP and NNp groups show a low level of vitamin D, the trend is quite different. While the latter shows a linearity of vitamin deficiency independently from the age, a phenomenon probably connected with chronicity of the disease, the PP group showed a decline of vitamin D strictly dependent on the age; the drops from low to very low based on the age are very clear and are suggestive of an acute pattern of the disease.

**Figure 2:**
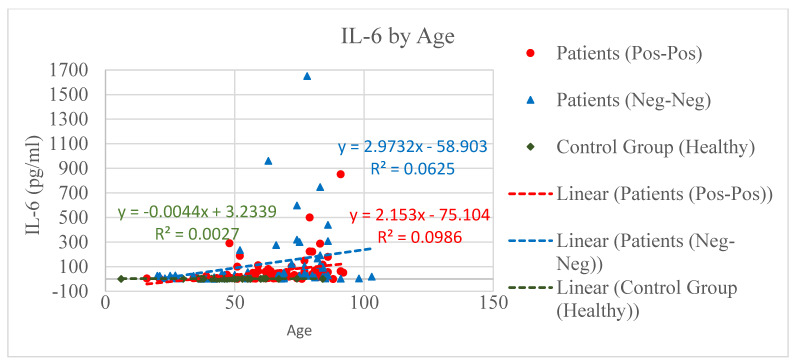
Scatter charts and regression lines for IL-6 by age for COVID-positive (PP), COVID-free patients (NNp), and the healthy group (NNh). It is a truncated IL-6 axis to show more details in the medium area of IL-6 concentration. IL-6 growth is seen in PP and NNp event consistent with an ongoing inflammatory process associated with age; the older the patient is, the higher the IL-6 is.

**Figure 3:**
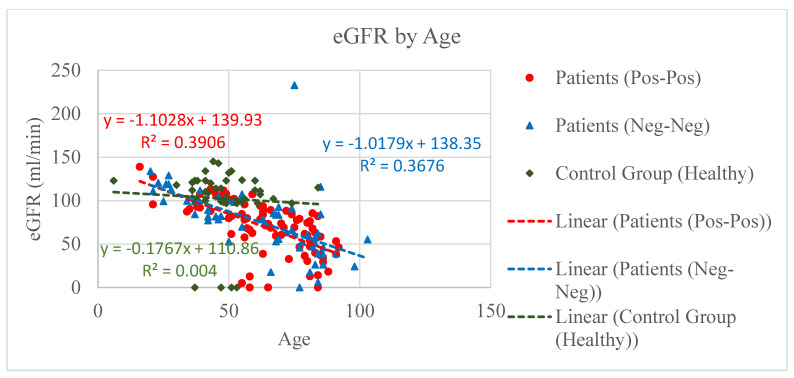
Scatter charts and regression lines for eGFR by age for COVID-positive (PP), COVID-free patients (NNp), and the healthy (NNh) group. The dotted eGFR axis shows more details for the medium area of eGFR concentration. This figure explicitly shows a significant decline of the eGFR level for COVID-positive and COVID-free NNp patients but no decline of the GFR level in the control group NNh. Nevertheless, the decline in the PP group is slightly higher than that of the NNp group.

**Figure 4:**
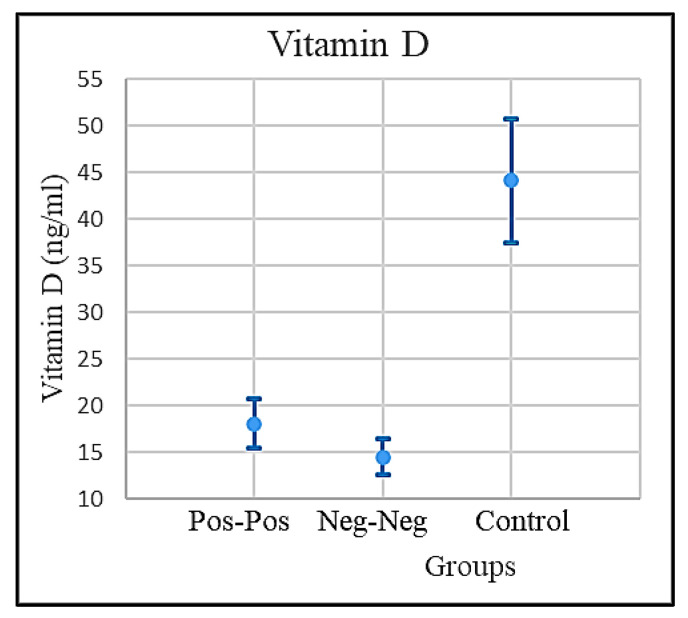
The vitamin D mean levels and the confidence intervals for COVID-positive, COVID-free patients, and the control groups.

**Figure 5:**
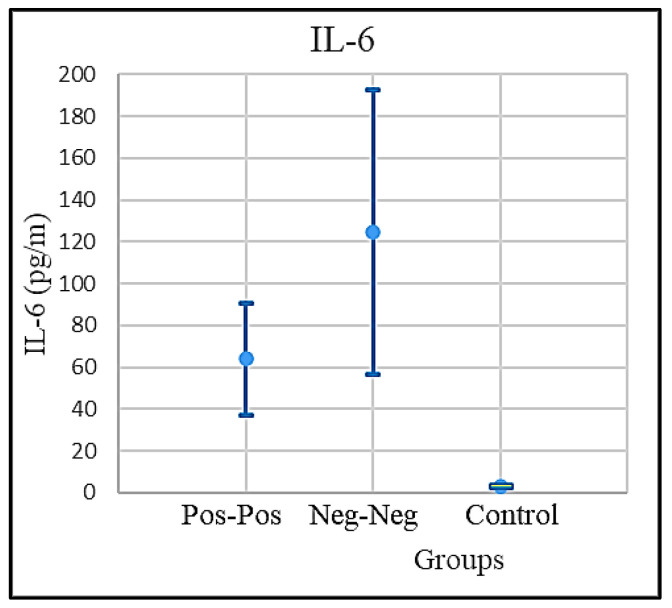
The IL-6 mean levels and the confidence intervals for COVID-positive, COVID-free patients, and the control groups.

**Figure 6:**
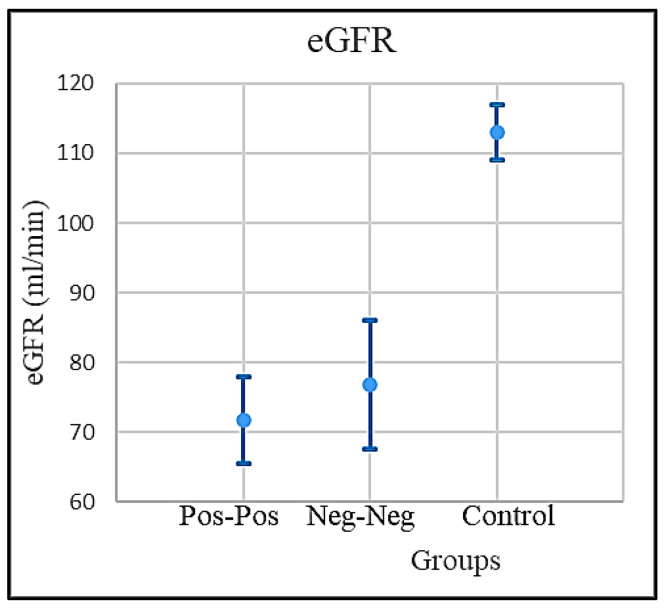
The eGFR mean levels and the confidence intervals for COVID-positive, COVID-free patients, and the control groups.

Three groups of people were designed and compared based on their vitamin D, IL-6, and eGFR concentration level. The first group was composed by COVID-positive patients (PP *n* = 80), positive to both nose–pharyngeal swab (RT-PCR) and CT scan (ground-glass opacity); the second group, the control group, included patients admitted to the hospital for other reasons than COVID (NNp *n* = 60) but negative to swab and CT scan; and the third group consisted of non-healthy individuals (NNh *n* = 43) Figure 1, Figure 2, Figure 3, Figure 4, Figure 5 and Figure 6.

The NNh group showed a normal concentration of vitamin D (around 50 ng/mL) compared to both PP and NNp groups that showed a significant low level of vitamin D. While the mean value of vitamin D level in the NNh group has to be considered within normal parameters, the mean value in the COVID-positive and NNp groups was visibly anomalous. The normal vitamin D level is about >30 ng/mL (sufficient) and >50 ng/mL (optimal). The vitamin D mean values and the respective confidence intervals are shown in Figure 6. Though both groups PP and NNp showed a low level of vitamin D, the trend resulted quite differently. The NNp’s outcomes revealed vitamin deficiency almost independently from the age, while the PP group’s results showed a decline of vitamin D strictly closely associated to the age; the drops from low to very low based on the age were very clear. However, same-age younger patients in the PP and NNp groups showed different level of vitamin D. The individuals in the PP group had a higher vitamin D level than those in the NNp group (Figure 1).

The IL-6 levels assessed in the three groups showed that both the PP and NNp groups manifested a significant increase of IL-6, an event strictly related to the patient age, while the IL-6 level in NNh was normal (<7 pg/mL) and practically independent from the age value (Figure 2).

Though the different levels of eGFR in both PP and NNp were not statistically significant, they confirmed an important decline in kidney functionality related either with age or to the grade of current illness; in the control group NNh, results showed only a shallow drop (Figure 3).

### 3.2. Statistical Findings

The mean values of vitamin D, IL-6, and eGFR in the COVID-positive, NNp, and the NNh groups, together with the respective confidence intervals, are shown in Table 1. The confidence intervals were calculated with the confidence level 95%, using Student’s *t*-test as shown in Figure 4. The mean values of IL-6 in the control group are normal. The IL-6 level in the COVID-positive group is nine times higher than normal, and patients in NNp, showed an extremely high IL-6 level, exceeding the normal level by eighteen times. The normal level of IL-6 < 7 pg/mL. The IL-6 mean values and the respective confidence intervals are shown in Figure 5.

The mean value of eGFR in the control group (NNh) was normal, while the eGFR level in both the PP and NNp groups was slightly below the normal. The normal level of eGFR is >90 mL/min. The eGFR mean values and the respective confidence intervals are shown in Figure 6.

### 3.3. Comparison of the Study Populations

The comparative analysis conducted on the differences of the mean values of vitamin D, IL-6, and eGFR in three groups—PP, NNp, and the control group, NNh—matched to the populations represented by these groups as samples (Figure 4, Figure 5 and Figure 6). 

The hypotheses about differences of the mean values of vitamin D, IL-6, and eGFR levels in PP, NNp, and the NNh were tested by using the Student’s *t*-test with the significance level alpha = 5%.

Three series of hypotheses were formulated regarding the difference of mean values: of vitamin D, IL-6, and eGFR in three groups PP, NNp, and NNh as independent samples.

1. Hypotheses about the difference of vitamin D in three groups:
  1.1. The hypothesis about the difference of vitamin D levels iin PP and NNp groups.
  1.2. The hypothesis about the difference of vitamin D levels iin the PP and NNh groups.
  1.3. The hypothesis about the difference of vitamin D levels in the NNp and NNh groups.
2. Hypotheses about the difference of vitamin D in three groups:
  2.1. The hypothesis about the difference of IL-6 levels iin the PP and NNp groups.
  2.2. The hypothesis about the difference of IL-6 levels iin the PP and NNh groups.
  2.3. The hypothesis about the difference of IL-6 levels iin the NNp and NNh groups.
3. Hypotheses about the difference of vitamin D in three groups:
  3.1. The hypothesis about the difference of eGFR levels in the PP and NNp groups.
  3.2. The hypothesis about the difference of eGFR levels in the PP and NNh groups.
  3.3. The hypothesis about the difference of eGFR levels in the NNp and NNh groups.

For all the above hypotheses the null and the alternative hypotheses are;

The null hypothesis:

**Hypothesis 1** **(H1).** *There is no difference in the respective parameter (vitamin D, IL-6, and eGFR) levels in the compared groups. The difference is statistically insignificant.*

The alternative hypothesis:

**Hypothesis 2** **(H2).** *There is a statistically significant difference in the respective parameter (vitamin D, IL-6, eGFR) levels in the compared groups.*

The hypotheses’ testing was conducted by using Student’s *t*-test with alpha = 5%. The test results are presented in Table 2, Table 3 and Table 4.

## 4. Discussion

We speculated the existence of the lung, heart, and kidney axis as main pathway for the COVID-19 disease progression mainly based on the existence of symmetrical anomalous markers, such as vitamin D, eGFR, and IL-6. The role of vitamin D or pro-hormone D in viral infection is a hot topic and matter of growing interest. Immunologically the focus is on D involvement in the entire cascade of host responses towards virus invasion. Recent findings have confirmed the D immunomodulatory effects and induction of autophagy, apoptosis, and even direct antiviral effects. There is a considerable variation in the prevalence of vitamin D deficiency across the world, mainly dependent on age; in COVID-19, elderly people and patients with metabolic comorbidities were the highest with fatality rates and were also those with lower serum of 25OHD levels [19,25,26,31]. The Seneca study revealed a mean 25OHD concentration of 10.4 ng/mL in elderly people aged 70 to 75 years in Spain. In this regard, experimental evidence indicates that vitamin D may inhibit IL-6, IL-1, IL-17, TNF-α, and IFN-γ by reducing the p38 MAP kinase activation in human monocytes/macrophages (M1 and neutrophils), enhancing the activity of T-regulatory cells (T-regs), Th2, M2, and IL-10 [31,34,35,36,37].

Thus, we speculated that the key pathogenic mechanism for SARS-CoV-2 to successfully grow could be eased by the low level of vitamin D, due to its role as crucial mediator of the Ang-II production within the RAS and ACE2/ACE2r mechanism. The whole sequelae of severe complications typical of COVID-19 disease begins via the downregulation of both ACE2/ACE2r expression, which is the main entrance for the virus to invade human cells, tissues, organs, and systems. The consequent Ang-II high accumulation drives to an uncontrolled hyper-inflammatory state that occurs under the overexpression of IL-6, a prerogative condition towards acute heart and renal failures especially witnessed in elderly people or in patients with previous metabolic comorbidities. 

**Figure 7:**
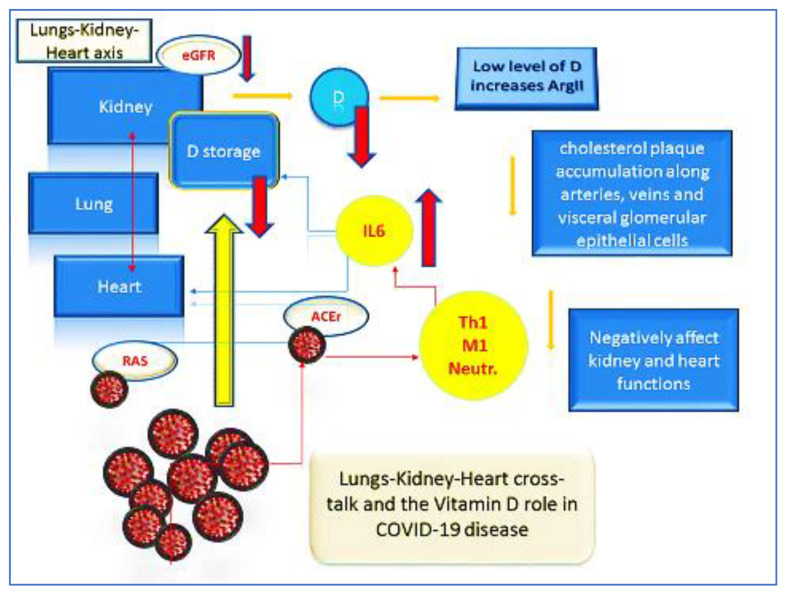
The regulation of acid–base equilibrium; modification of partial pressure of carbon dioxide, oxygen, and bicarbonate concentration; and the control of blood pressure, fluid homeostasis, and systemic blood supply all closely depend on renal and heart pulmonary activities. SARS-CoV-2 dangerously spreads prevalently, due to the low level of vitamin D as crucial mediator of the Ang-II production within the RAS and the angiotensin-converting enzyme 2 receptor (ACE2R) and the angiotensin-converting enzyme 2 mechanism (ACE2/ACE2r). The entire sequence of COVID-19-related complications starts via the downregulation of ACE2r expression as the SARS-CoV-2 main entrance. The Ang-II high accumulation induces uncontrolled hyper-inflammatory responses led by the IL-6 scenario that has been seen as prerogative cause of acute heart and renal failures particularly witnessed in elderly people or in patients with preexisting metabolic comorbidities. The totality of adverse effects related to Ang-II eases cholesterol plaque accumulation along vases and visceral glomerular epithelial cells (podocytes) that provoke systemic and glomerular hypertension or cause ischemia-induced renal injury, which results in kidney and cardio-vascular failures (figure by C. Gargiulo Isacco).

As mentioned above, the adverse effects of Ang-II may enhance an uncontrolled cholesterol plaque accumulation along vessels and visceral glomerular epithelial cells (podocytes) that, in turn, aggravates systemic and glomerular hypertension, favoring the insurgence of ischemic-induced renal injuries, leading to either kidney or cardio-vascular failures (Figure 7) [1,2,3]. The Ang-II contribution to the pathophysiology of cardiac and kidney failure is mainly based on the following observations: (1) Ang-II is produced within the myocardium and renal cortex, (2) Ang-II is activated within the failing hypertrophic heart and overproduced in hypertrophic kidneys, and (3) the pharmacological inhibition of the RAS and Ang-II in either animal models or in patients with a hypertrophic heart and injured kidney showed high effectiveness. In this perspective, few authors proposed kidneys low level of D production as key factor of COVID-19 related super-inflammatory mode and organ failures [1,2,3,38,39,40].

The complete shutdown of vitamin D synthesis would consequently enhance an uncontrolled reaction over accumulation of Ang-II that, in turn, scatters the high viability of pro-inflammatory cytokine and interleukins, such as IL-6, followed by a sudden regression of kidney, heart, and lung axis functionality (7). 

We found that vitamin D deficiency, high level of IL-6 and low level of eGFR were highly indicative in the COVID-19 tropism, the differences of the mean levels of vitamin D, IL-6, and eGFR obtained by comparing the three groups were statistically significant. The data suggested that vitamin D was correlated with disease typology either COVID-19 or other typical inflammatory type diseases, acute or chronic, as it was expressed by PP and NNp groups, indicating its role in COVID-19 acumination and pathogenesis (*p* > 0.05).

Both IL-6 and eGFR were also statistically significant. The data showed that, once the PP and NNp groups were compared with the PPh, IL-6 and eGFR were also indicative of the grade of severity of different diseases (NNp) but also revealed common patterns that make COVID-19 ((*p* > 0.05). Compared to the inflammatory markers used in the clinic routine, we found a positive correlation between IL-6 and the grade of infection. However, despite the ubiquitous role of IL-6 in different types of infections, when we assessed the predictive value, this marker, IL-6, behaves as a valued predictor of COVID-19 progression. In fact, in a binary logistic regression, IL-6 level resulted to be a significant predictor of the non-survivors group, when compared to age and other inflammatory markers such as CRP, VES, or D-dimer. 

Age eventually plays a critical role in keeping a proper and balanced immune response. The data clearly showed that levels of vitamin D, eGFR, and IL-6 are inversely correlated; the older the individual is, the lower vitamin D and eGFR, and the higher the IL-6 (Table 3 and Table 4).

Therefore, a forthcoming therapy would advocate the rationale use of vitamin D. However, based on our clinical experience, we propose that this strategic approach would include a combined approach that allow us to act on the immune system, on the respiratory system, and on the nervous system, contributing to a better respiration via brainstem centers and phrenic nerve. For instance, the use of vitamin D, vitamin K, and erythropoietin (EPO) showed to modulate the ventilator response in the locus coeruleus to CO_2_ in rats, positively adjusting the hypercapnia-induced hyperpnoea [3,40,41,42]. Vitamin D was shown to mediate the intracellular oxidative stress, increasing superoxide dismutase 1 and 2 (SOD1-2) (as seen in cancer hypoxic cancer microenvironment). The EPO-mediated regulation of the central respiratory command, which involves both MEK1/2 and PI3K pathways, was seen as crucial for phrenic motor facilitation, suggestive of a spinal plasticity in respiratory motor control in prolonged poor oxygen condition. On the other hand, the vitamin K not only allows the activation of D via the hydroxylation mechanism, but also prevents cell mitochondrial dysfunction, restores oxidative phosphorylation and the aerobic glycolysis, and eventually supports the cleansing hypoxic microenvironment typical of tissues analyzed in cancers and lung and cardiovascular necrosis due to massive thromboembolism [3,4,5,41,43,44,45,46,47,48] (Figure 7). 

To summarize, heart, lung, and kidney functions are closely related, especially if one sees the whole picture under the perspective of emergency medicine. The regulation of acid–base equilibrium; modification of partial pressure of carbon dioxide, oxygen, and bicarbonate concentration; and the control of blood pressure, fluid homeostasis, and systemic blood supply all closely depend on renal and heart pulmonary activities. It follows that the grade severity of the COVID-19 would depend on age and pre-existing diseases, as well as on an over-consumption of stored vitamin D within the body in response to SARS-CoV-2 invasion via RAS/ACEr mechanism. With this concept in mind, it follows that any therapeutic approach should include a flexible approach which previews the use of multiple agents, especially in those individuals at high risk, such as the elderly and patients with metabolic–chronic comorbidities [40,49,50,51,52,53].

## 5. Conclusions

To conclude, we are well aware that a further study with a larger sample size is needed to confirm direct evidence of the effect of vitamin D on COVID-19 disease. Vitamin D deficiency was positively associated with the elevated IL-6 and decreased eGFR, especially in the PP study group. Furthermore, this study weighed our proposed hypothesis indicating vitamin D, eGFR, and IL-6 as strong markers in the COVID-19 disease-monitoring phase; meanwhile, they were also signifying the concrete existence of a kidney, heart, and lung axis.

## Figures and Tables

**Table 1:** Mean values of vitamin D, IL-6, and eGFR for three groups, together with their respective confidence intervals with the confidence level 95%.

	Groups	Mean	±δ	Confidence Interval	
Vitamin D (ng/mL)	PP Patients	18.0475	2.639475	15.40802	20.68698
	NNp	14.49355	1.871572	12.62198	16.36512
	NNh (Healthy)	44.0986	6.599012	37.49959	50.69762
IL-6 (pg/m)	PP Patients	64.10633	26.77383	37.3325	90.88016
	NNp	124.5258	68.18824	56.33757	192.714
	NNh (Healthy)	3.023256	0.337694	2.685562	3.36095
eGFR (mL/min)	PP Patients	71.71623	6.263475	65.45276	77.97971
	NNp	76.79361	68.18824	8.605366	144.9818
	NNh (Healthy)	112.9769	4.122511	108.8544	117.0994

**Table 2:** The Student’s *t*-test for differences of the vitamin D mean values in PP vs. NNp, PP vs. NNh, and PP vs. NNh groups; the differences of the mean levels of vitamin D in all three groups are statistically significant. The differences of the mean levels of vitamin D in all three groups are statistically significant.

Vitamin D (ng/mL)			
	PP	NNp	NNh
Diff of means =	3.55	26.05	29.61
Sp =	10.20	15.90	14.92
1/sqrt(np) =	0.17	0.19	0.20
df =	140	121	103
t-cr =	1.98	1.98	1.98
t-score =	2.06	8.67	10.00
Test is	Significant	Significant	Significant

**Table 3:** The Student’s *t*-test for differences of the IL-6 mean values in PP vs. NNp, PP vs. NNh, and NNp vs. NNh groups. The difference of the mean levels of IL-6 in COVID-positive (PP) NNp groups is statistically insignificant, but the difference of the mean values of IL-6 in the NNh group and the PP and NNp groups was statistically significant.

IL-6 (pg/m)			
	PP	NNp	NNh
Diff of means =	60.42	61.08	121.50
Sp =	198.93	97.24	206.60
1/sqrt(np) =	0.17	0.19	0.20
df =	140	121	103
t-cr =	1.98	1.98	1.98
t-score =	1.80	3.32	2.96
Test is	Insignificant	Significant	Significant

**Table 4:** The Student’s *t*-test for differences of the eGFR mean values in PP vs. NNp, PP vs. NNh, and NNp vs. NNh groups. The difference of the mean levels of eGFR in PP, NNp, and NNh groups is statistically insignificant, but the difference of the mean values of eGFR in the NNh compared to both the PP and NNp groups was statistically significant.

eGFR (mL/min)			
	PP	NNp	NNh
Diff of means =	5.08	41.26	36.18
Sp =	32.27	24.09	29.69
1/sqrt(np) =	0.17	0.19	0.20
df =	140	121	103
t-cr =	1.98	1.98	1.98
t-score =	0.93	9.06	6.14
Test is	Insignificant	Significant	Significant

## Data Availability

All experimental data to support the findings of this study are available contacting the corresponding author upon request.
